# Hepatitis B Virus Seropositivity Is a Poor Prognostic Factor of Pediatric Hepatocellular Carcinoma: a Population-Based Study in Hong Kong and Singapore

**DOI:** 10.3389/fonc.2020.570479

**Published:** 2020-11-20

**Authors:** Anthony P. Y. Liu, Shui-Yen Soh, Frankie W. C. Cheng, Herbert H. Pang, Chung-Wing Luk, Chak-Ho Li, Karin K. H. Ho, Edwin K. W. Chan, Albert C. Y. Chan, Patrick H. Y. Chung, Miriam S. Kimpo, Summaiyya H. Ahamed, Amos Loh, Alan K. S. Chiang

**Affiliations:** ^1^Department of Paediatrics and Adolescent Medicine, The University of Hong Kong, Queen Mary Hospital, Hong Kong, Hong Kong; ^2^Department of Pediatrics, KK Women’s and Children’s Hospital, Singapore, Singapore; ^3^Department of Paediatrics, Prince of Wales Hospital, Hong Kong, Hong Kong; ^4^School of Public Health, The University of Hong Kong, Hong Kong, Hong Kong; ^5^Department of Paediatrics, Queen Elizabeth Hospital, Hong Kong, Hong Kong; ^6^Department of Paediatrics and Adolescent Medicine, Tuen Mun Hospital, Hong Kong, Hong Kong; ^7^Department of Paediatrics and Adolescent Medicine, Princess Margaret Hospital, Hong Kong, Hong Kong; ^8^Department of Surgery, Prince of Wales Hospital, Hong Kong, Hong Kong; ^9^Department of Surgery, The University of Hong Kong, Queen Mary Hospital, Hong Kong, Hong Kong; ^10^Division of Pediatric Hematology/Oncology & Bone Marrow and Cord Blood Transplantation, University Children’s Medical Institute, National University Hospital, Singapore, Singapore; ^11^Department of Diagnostic and Interventional Imaging, KK Women’s and Children’s Hospital, Singapore, Singapore; ^12^Department of Paediatric Surgery, KK Women’s and Children’s Hospital, Singapore, Singapore

**Keywords:** hepatocellular carcinoma, pediatric, hepatitis B virus, vaccination, surveillance

## Abstract

**Background:**

Hepatocellular carcinoma (HCC) is a rare hepatic malignancy in children. Hepatitis B virus (HBV) infection is a key predisposing factor in endemic regions but its impact on outcome has not been studied. We aim to evaluate the prognostic implication of HBV seropositivity and role of cancer surveillance in children with HCC from East Asian populations with national HBV vaccination.

**Methods:**

Review of population-based databases for patients (< 18 years old) diagnosed with HCC from 1993 to 2017 in two Southeast Asian regions with universal HBV vaccination (instituted since 1988 and 1987 in Hong Kong and Singapore, respectively).

**Results:**

Thirty-nine patients were identified (Hong Kong, 28; Singapore, 11). Thirty were male; median age at diagnosis was 10.8 years (range, 0.98–16.6). Abdominal pain was the commonest presentation while five patients were diagnosed through surveillance for underlying condition. Alpha-fetoprotein was raised in 36 patients (mean, 500,598 ng/ml). Nineteen had bilobar involvement, among the patients in whom pretreatment extent of disease (PRETEXT) staging could retrospectively be assigned, 3 had stage I, 13 had stage II, 4 had stage III, and 11 had stage IV disease. Seventeen had distant metastasis. HBsAg was positive in 19 of 38 patients. Two patients had fibrolamellar HCC. Upfront management involved tumor resection in 16 (liver transplantation, 2), systemic chemotherapy in 21, interventional procedures in 6 [transarterial chemoembolization (TACE), 5, radiofrequency ablation (RFA), 1], and radiotherapy in 4 (selective internal radiation, 3, external beam radiation, 1). Five-year event-free survival (EFS) and overall survival (OS) were 15.4 ± 6.0 and 26.1 ± 7.2%, respectively. Patient’s HBsAg positivity, metastatic disease and inability to undergo definitive resection represent poor prognostic factors in univariate and multivariable analyses. Patients diagnosed by surveillance had significantly better outcome.

**Conclusion:**

Pediatric HCC has poor outcome. HBV status remains relevant in the era of universal HBV vaccination. HBV carrier has inferior outcome and use of surveillance may mitigate disease course.

## Introduction

Hepatocellular carcinoma (HCC) is an uncommon but aggressive malignancy in the pediatric age group (1). Disease epidemiology varies among populations and is influenced by differences in underlying etiologies (2). Chronic hepatitis B virus (HBV) infection is endemic in Southeast Asia and has been the most important contributing factor to pediatric HCC in these regions through vertical maternal-fetal transmission of the virus. In particular, Hong Kong and Singapore are two developed areas with similar population structure and prevalence of chronic HBV carrier at 5–6% (3, 4). Universal HBV vaccination for newborns and infants have been instituted in Hong Kong and Singapore since 1988 and 1987, respectively. Decrease in incidence of pediatric HCC has previously been demonstrated by the Taiwanese group, but the condition is likely to remain relevant in the East due to vaccine failure and postnatal horizontal transmission (5–7). In addition, the prognostic significance of HBV seropositivity in pediatric HCC as well as the impact of surveillance in children with known chronic liver conditions have not been well described. Here we report the outcome of pediatric HCC from Hong Kong and Singapore exploiting population-based databases accruing childhood cancer data since the 1990s. Disease characteristics between patients with and without HBV infection are compared and the impact of HBV seropositivity as well as cancer surveillance on patient outcome is evaluated.

## Materials and Methods

### Study Cohort

We reviewed population-based pediatric cancer data in Hong Kong and Singapore for patients diagnosed with histologically proven HCC under the age of 18 years. The Hong Kong cohort was based on data prospectively contributed by all five local pediatric oncology units (Queen Mary Hospital, Prince of Wales Hospital, Tuen Mun Hospital, Queen Elizabeth Hospital, Princess Margaret Hospital) through the Hong Kong Paediatric Haematology Oncology Study Group between 1996 and 2017 and the Singaporean cohort was based on data contributed by the two major pediatric oncology units (KK Women’s and Children’s Hospital, National University Health System) between 1993 and 2017. Available data on patients’ demographics, clinical presentation, baseline biochemical and alpha-fetoprotein (AFP) levels, extent of disease [lobar involvement, portal vein (PV) involvement, metastatic disease], histological findings, patients’ and maternal HBV serology [hepatitis B surface antigen (HBsAg), hepatitis B surface antibody (anti-HBs Ab)], patients’ HBV vaccination status, other etiological factors, treatment received [surgery, chemotherapy, interventional procedure, radiation therapy, liver transplantation (LT)] and outcome were retrieved from paper and electronic patient records. Pretreatment extent of disease (PRETEXT) staging was retrospectively assigned based on review of imaging reports according to the 2017 PRETEXT criteria (8).

### Incidence Estimation

Incidence rates of pediatric HCC in Hong Kong and Singapore between 1996 and 2017 were estimated in relation to the resident population from national census data. Patients who were non-residents were excluded from this analysis (3 from the Hong Kong cohort). Population size (0–<18 years of age) was determined and extrapolated where necessary from government census data (Hong Kong, 1996, 2001, 2006, 2011, 2016; Singapore, 1996–2017).

### Statistical Analysis

Average and dispersion of variables were summarized as median and range or mean and standard deviation (SD). Using the Kaplan-Meier method, overall survival (OS) was estimated based on the duration between the date of diagnosis and the date of death or last follow‐up (censored), while event‐free survival (EFS) was estimated based on the duration between the date of diagnosis and the date of relapse, death from any cause, or last follow‐up (censored). Log-rank test and Cox regression were performed to evaluate the impact of demographic (age, sex, year of diagnosis), clinical [presenting symptoms, patient HBsAg serostatus, AFP, alkaline phosphatase (ALP), alanine transferase (ALT), extent of disease including lobar involvement, PV involvement, distant metastasis and PRETEXT stage], and treatment variables [extent of surgery (ability to undergo tumor resection, including transplantation, *versus* biopsy only), chemotherapy use, radiotherapy use, interventional radiological procedures] on patient outcome in univariate and multivariable analyses. Differences in presentation and clinical parameters between patients with and without HBsAg positivity were studied by chi-square or Fisher’s exact test. Statistical analysis was performed with R version 3.5.0. This study was approved by the respective Institutional Review Boards with requirement of informed consent waived.

## Results

### Patient Demographics and Disease Incidence

Thirty-nine patients were identified (Hong Kong, 28; Singapore, 11; **Table 1**, **Supplementary Table S1**. Thirty patients were male (76.9%) and median age at diagnosis was 10.8 years (mean, 10.2, range, 0.98-16.6). Nineteen patients (48.7%) were diagnosed before 2002 and 20 patients (51.2%) were diagnosed in or after 2002. Crude incidence rates for the Hong Kong and Singapore cohorts were 0.90 (SD, 1.12) and 0.49 (SD, 0.60) per million per year, respectively between 1996 and 2017 (**Supplementary Table S2**).

**Table 1:** Demographics, biochemical, radiographical characteristics of the entire cohort and comparison according to hepatitis B surface antigen (HBsAg) serostatus.

** **	Entire cohort		HBsAg +		HBsAg -		P value (HBsAg + *vs.* −)
	N	%	N	%	N	%	
**Total**	39	100	19	100	19	100	
							
**Sex**							0.002
**Male**	30	77	19	100	10	53	
**Female**	9	23	0	0	9	47	
							
**Age of diagnosis (years) (mean ± SD)**	10.2 ± 4.1	–	10.8 ± 3.7	–	9.8 ± 4.5	–	0.450
							
**Period of diagnosis**							1
**1993–2001**	19	49	9	47	9	47	
**2002–2017**	20	51	10	53	10	53	
** **							
**ALP (IU/L)** **(mean ± SD)**	267 ± 195	–	234 ± 97	–	297 ± 251	–	0.354
**ALT (IU/L)** **(mean ± SD)**	287 ± 1,151		85 ± 80		501 ± 1,648		0.314
**AFP (ng/ml)** **(mean ± SD)**	500,598 ± 1,350,405	–	662,453 ± 1,821,612	–	338,743 ± 612,309	–	0.471
** **							
**Lobe involvement**							0.540
**Left**	6	15	2	11	4	21	
**Right**	11	28	5	26	6	32	
**Bilateral**	19	49	11	58	8	42	
**NA**	3	8	1	5	1	5	
							
**Portal vein involvement**							0.500
**Yes**	7	18	5	26	2	11	
**No**	27	69	13	68	14	74	
**NA**	5	13	1	5	3	16	
							
**Distant metastasis**							0.880
**Yes**	17	44	9	47	8	42	
**No**	20	51	9	47	11	58	
**NA**	2	5	1	5	0	0	
							
**Regional lymph node**							0.637
**Involved**	4	10	1	5	3	16	
**Not involved**	33	85	17	89	16	84	
**NA**	2	5	1	5	0	0	
							
**Definitive resection**							0.255
**Yes**	16	41	6	32	10	53	
**No**	21	54	13	68	8	42	
**NA**	2	5	0	0	1	5	

AFP, alpha fetoprotein; ALP, alkaline phosphatase; HBsAg, hepatitis B surface antigen; NA, not available; SD, standard deviation.

### Baseline Disease Characteristics

The most common presenting symptoms were abdominal pain (N = 21), distension (N = 12), and a palpable mass (N = 9). Five patients were diagnosed by surveillance using serum AFP performed due to known HBV carrier status (N = 3) or underlying chronic liver disease (CLD) (N = 2). At diagnosis, serum AFP was elevated in 36 of 38 patients (94.7%; mean, 500,598 ng/ml; range, 169–7,999,999; missing data in 1) while AFP was normal in two patients with fibrolamellar HCC. Hyperbilirubinemia, raised alkaline phosphatase (ALP), raised alanine aminotransferase (ALT) were present in 4 of 32 [12.5%, abnormal value mean, 325 µmol/L (range, 165–558)], 7 of 32 [21.9%, abnormal value mean, 535 IU/L (range, 362–1,060)], and 19 of 35 [54.3%, abnormal value mean, 502 IU/L (range, 62–6,883)], respectively.

Radiographically, 6 (16%), 11 (30%), and 19 (51%) of 37 patients had involvement of the left, right, and both hepatic lobes, respectively. PRETEXT staging was I in 3 (8%) patients, II in 13 (33%), III in 4 (10%), IV in 11 (28%), not available in 8 (21%). PV involvement was present in 7 of 34 (20.5%). Seventeen of 37 (45.9%) had distant metastasis at diagnosis, of which 16 patients had metastases to the lungs among whom one patient additionally had skeletal metastasis and two had regional lymph node metastasis. The one remaining patient had metastases to the chest wall, skeleton, and regional lymph nodes. Tumors detected by surveillance were all localized (p = 0.049) and associated with significantly lower serum AFP levels (mean 3,063 *vs.* 575,982 ng/ml; p = 0.029; **Table 2**).

**Table 2:** Clinical characteristics, treatment, and outcome for patients diagnosed through surveillance.

Patient no.	Sex	Age (years)	Underlying condition	Lobe involved	LN involvement	Distant metastasis	Hepatic vein involvement	ALP (IU/L)	AFP (ng/ml)	Treatment	Disease status	Duration of FU (years)
3	Male	7.1	Neonatal hepatitis with cirrhosis	Left	No	No	No	1,060	4,278	Complete resection → C5V → carboplatin → hepatic relapse → LT	NED	20.80
24	Male	12.6	HBsAg +	Right	No	No	No	326	169	Complete resection	NED	6.96
34	Male	1.1	PFIC	Bilobar	No	No	No	307	8,503	Biopsy → LT	NED	19.65
36	Male	5.2	HBsAg +	NA	No	No	No	NA	644	Complete resection	NED	4.01
37	Male	7.4	HBsAg +	Right	No	No	No	362	1,719	Complete resection → hepatic relapse → TACE	DOD	11.26

AFP, alpha fetoprotein; ALP, alkaline phosphatase; C5V, cisplatin+fluorouracil+vincristine; DOD, died of disease; FU, follow-up; HBsAg, hepatitis B surface antigen; LN, lymph node; LT, liver transplantation; NA, not available; NED, no evidence of disease; Progressive Familial Intrahepatic Cholestasis; TACE, transarterial chemoembolization.

### Chronic Hepatitis B Virus Infection and Other Etiological Factors

**Figure 1:**
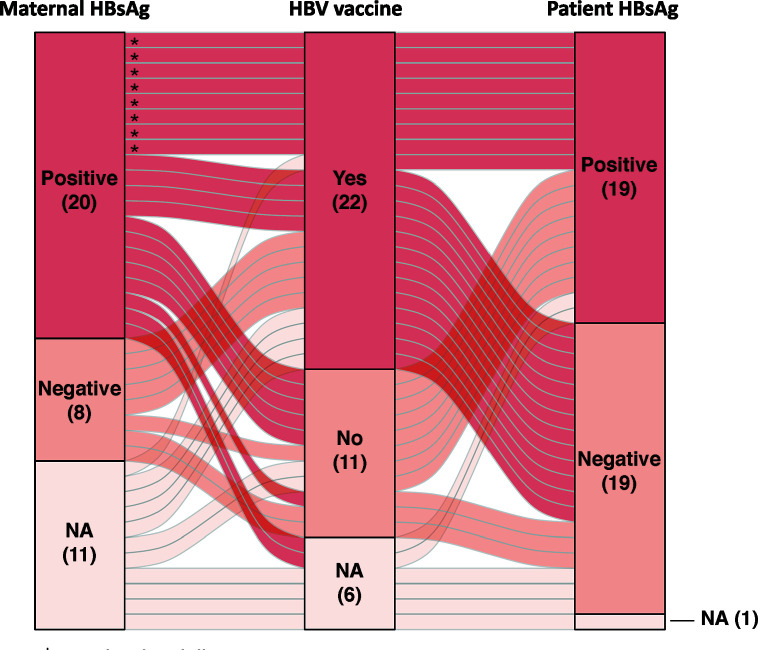
Alluvial plot depicting the patients’ hepatitis B virus (HBV) serology, maternal HBV serology, and HBV vaccination status in our cohort (* patients with vaccine failure). HBsAg, hepatitis B surface antigen; NA, not available.

Nineteen of 38 patients (50%) were HBsAg seropositive with 15 born to HBsAg positive mothers, 1 to HBsAg negative, and 3 to mothers with unknown HBV serostatus (**Figure 1**, **Supplementary Figure S1**). All 19 HBsAg positive patients were males, while male to female ratio for HBsAg negative patients was 10:9 (**Table 1**). Other demographic and clinical characteristics, including age of diagnosis, period of diagnosis, serum AFP level, extent of disease and resectability, did not differ significantly between HBsAg positive or negative patients. Thirty-three patients had available vaccination data, of which 22 (66.7%) completed HBV vaccination in infancy; whereas 7 of 16 patients born to HBsAg positive mothers received HBIg. Nine patients were HBsAg positive despite completing HBV vaccination with five having received HBIg. Cirrhosis was documented in 6 of the HBsAg positive patients. Among patients who were HBsAg negative, three had cirrhosis due to biliary atresia, progressive familial intrahepatic cholestasis and neonatal hepatitis, respectively.

### Treatment, Outcome, and Prognostic Factors

**Figure 2:**
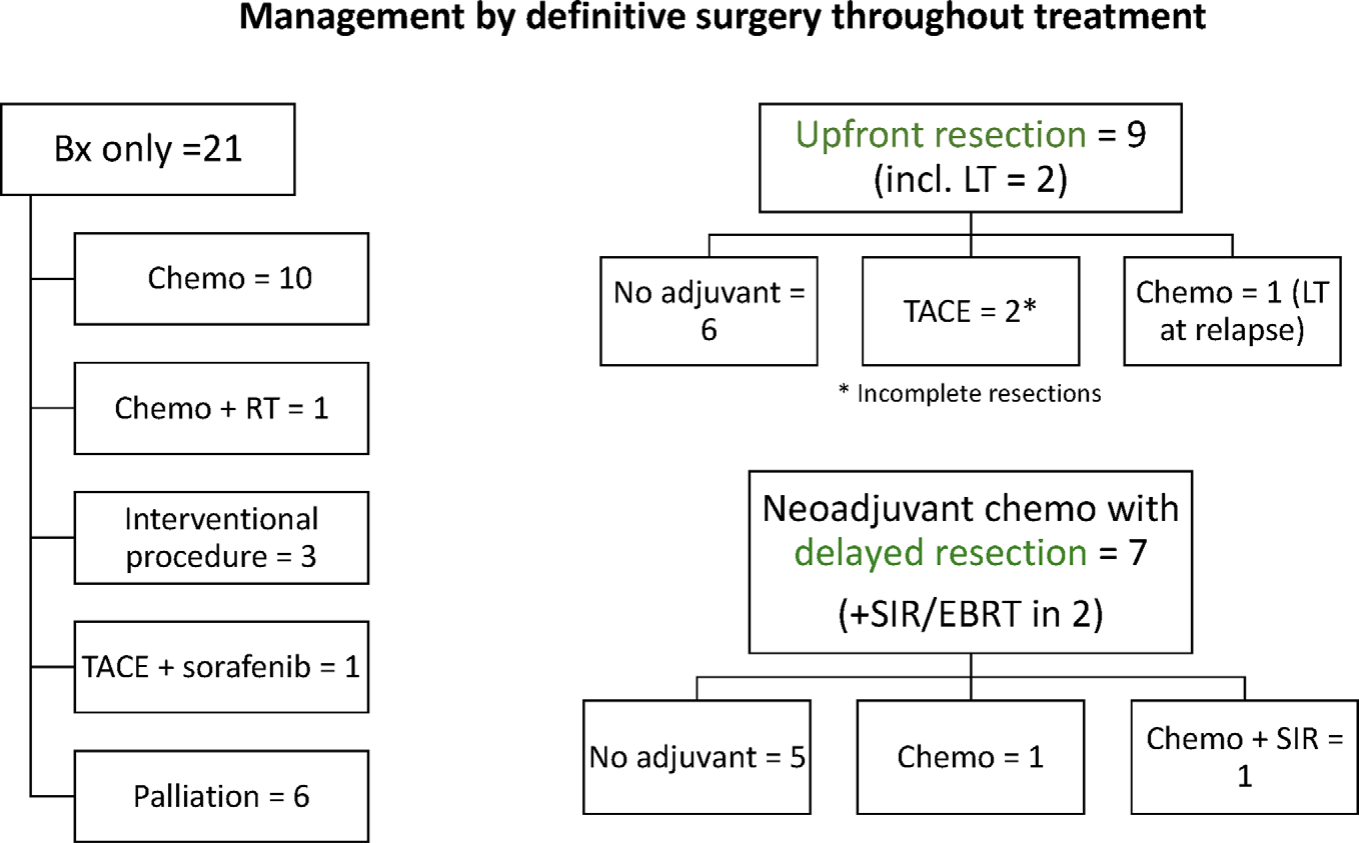
Summary of neoadjuvant and adjuvant treatment regimens received by patients in our cohort according to resectability (two patients with missing or incomplete treatment details excluded).

**Figure 3:**
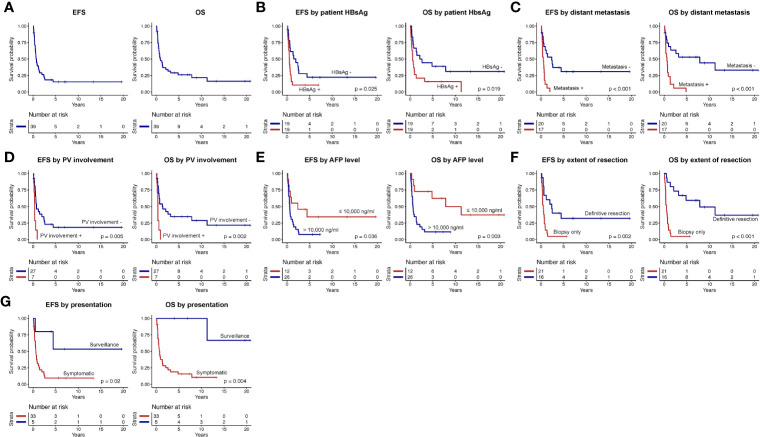
Event-free survival (EFS) and overall survival (OS) of **(A)** the entire study cohort, according to **(B)** patients’ hepatitis B surface antigen (HBsAg) serostatus, **(C)** presence or absence of distant metastasis, **(D)** portal vein (PV) involvement, **(E)** alpha-fetoprotein (AFP) level, **(F)** extent of resection, and **(G)** mode of presentation (surveillance *versus* symptomatic).

Treatment regimens received by patients in our cohort were heterogeneous (**Figure 2**). Extent of surgery consisted of biopsy only in 21 and definitive resection in 16: complete resection in 11, partial resection in 2, and LT in 3. Adjuvant therapy included systemic therapy in 21 patients, common chemotherapeutic agents included cisplatin, 5-fluorouracil, vincristine, and doxorubicin. Interventional radiological procedures were conducted in six patients (transarterial chemoembolization, five; radiofrequency ablation, one), and radiotherapy in four (selective internal radiation therapy, three, external beam radiotherapy, one). Nine patients were alive without disease at last follow-up (duration of follow, median, 7.3 years, range 0.2–20.8 years), while 30 patients died of disease progression. 5-year EFS and OS rates were 15.4 ± 6.0 and 26.1 ± 7.2%, respectively (**Figure 3**). Significant predictors for inferior survival included HBsAg positivity (EFS, p = 0.025; OS, p = 0.019), metastatic disease (EFS and OS, p < 0.001), PV involvement (EFS, p = 0.005; OS, p = 0.002), AFP level >10,000 ng/ml (EFS, p = 0.036; OS, p = 0.003), and patients who received biopsy only instead of definitive resection (EFS, p = 0.002; OS, p < 0.001). Patients who were diagnosed by surveillance had significantly better outcome (EFS, p = 0.02; OS, p = 0.004). On multivariable analysis, HBsAg positivity (EFS, p = 0.045; OS, p = 0.027) and extent of resection (biopsy *vs.* definitive resection, OS, p = 0.021) remained as significant predictors (**Table 3**).

**Table 3:** Multivariable Cox regression analysis on predictors for event-free survival (EFS) and overall survival (OS).

	EFS			OS		
Characteristics	Hazard ratio	95% CI	*P*	Hazard ratio	95% CI	*P*
**Patient HBsAg**						
Positive	2.51	1.02–6.17	0.045	2.88	1.13–7.39	0.027
Negative	Reference			Reference		
**Metastasis**						
Yes	2.43	0.91–6.47	0.077	2.82	0.97–8.26	0.058
No	Reference			Reference		
**PV involvement**						
Yes	2.29	0.78–6.78	0.134	2.02	0.69–5.91	0.199
No	Reference			Reference		
**Alpha fetoprotein**						
> 10,000 ng/ml	1.79	0.54–4.92	0.341	1.82	0.51–6.45	0.356
≤ 10,000 ng/ml	Reference			Reference		
**Definitive resection**						
Yes	0.61	0.22–1.67	0.333	0.24	0.07–0.81	0.021
No	Reference			Reference		
**Presentation**						
By surveillance	0.39	0.06–2.62	0.332	0.16	0.02–1.80	0.139
Symptomatic	Reference			Reference		

HBsAg; hepatitis B surface antigen; CI; confidence interval; EFS, event-free survival; OS, overall survival.

## Discussion

Due to its rarity, pediatric HCC has largely been understudied and the best management strategy for this aggressive condition is yet to be defined. Etiologies that predispose to the development of HCC in children vary geographically and ethnically, including mainly inborn errors of metabolism, biliary atresia and chronic viral hepatitis (9). Chronic HBV infection, in particular, is endemic in Asia and underlies a significant proportion of pediatric HCC reported in the region (10–13). Reducing the viral load in HBV positive mothers through antenatal anti-viral treatment, and universal newborn vaccination against HBV since the 1980s has reduced the risk of perinatal vertical transmission and is associated with decreasing incidence of pediatric HCC in the region (14–16). Study of 1,509 pediatric and young adult patients with HCC from the Taiwan registries revealed a two-third risk reduction for developing HCC between ages 6 and 19 years in individuals who had received HBV vaccination (13). Nonetheless, 1–11.8% of infants born to mothers with chronic HBV infection will develop breakthrough HBV infection despite vaccination, especially with incomplete vaccination schedule or mother with positive HBeAg (7, 17). Also, vaccination coverage is still not universal in many other countries in East Asia (18). Advancing our understanding on pediatric HCC in the context of chronic HBV infection therefore remains pertinent from a global perspective.

The outcome of pediatric HCC is much worse than that of hepatoblastoma (1, 19–21). Earlier Intergroup (INT-0098) and International Society of Paediatric Oncology (SIOP) (SIOPEL1) studies reported 5-year EFS of 19 and 17%, respectively, similar to the EFS observed in our cohort (15%) (1, 19). Resectability consistently emerged as the key prognostic factor in these patients, which was governed by the presence or absence of metastatic and/or multifocal disease, and in turn reflected by staging with the Evans or Pretreatment Extent of Disease (PRETEXT) system. In INT-0098 study, patients who received upfront complete resection were randomized to four cycles of adjuvant chemotherapy (CDDP/5-FU/VCR or CDDP/DOXO). Those with Evans Stage I HCC had 5-year EFS of 88% *versus* 8% and 0% for patients with stage III and IV disease, respectively. In SIOPEL1 where neoadjuvant chemotherapy (CDDP/DOXO) with delayed resection was adopted, metastasis significantly predicted both EFS and OS while PRETEXT staging was also significantly associated with OS (1, 19). In keeping with the above, we observed better outcome in patients with localized disease and for those who received definitive resection rather than diagnostic biopsy only upfront. Remarkably, we identified HBV seropositivity as a significant predictor of inferior survival by both univariate and multivariable analyses. In previous cohorts of pediatric HCC from Asia, disease etiology had been ascribed to chronic HBV infection which occurred at rates of between 71 and 100%. However, we have observed for the first time an association between HCC survival rates and HBV serostatus (10, 12). The lack of divergence in outcome in previous studies might be explained by the overall dismal prognosis in these cohorts (5-year OS 4 and 15.8%) (10, 12). The prognostic value of HBV serostatus is further substantiated by a lack of significant differences in potential confounders such as disease extent and resectability between the two groups. While the optimal treatment approach for pediatric HCC beyond maximally safe resection is yet to be defined, patients with underlying chronic HBV infection should be considered as a high-risk subgroup in the design of future trials enrolling patients from endemic regions.

Antenatal anti-viral treatment, HBV vaccination and HBIg prophylaxis represent the mainstay of preventing vertical viral transmission and subsequent risk for HCC development in newborns to mothers who are HBV carriers. Infants with breakthrough HBV infection despite the above might benefit from additional measures for early detection of tumor development. Surveillance for HCC through interval ultrasonography with or without serum AFP is recommended for adults with chronic liver disease including HBV infection although its role for the corresponding pediatric population is undefined (22, 23). Despite its inconsistent application, patients diagnosed through surveillance in our cohort had significantly better outcome. This included patients with HBV infection, progressive familial intrahepatic cholestasis (PFIC), and cirrhosis due to neonatal hepatitis. Compared with patients diagnosed after symptomatic presentation, disease detected by surveillance appeared to be less extensive and was associated with lower AFP levels. The universal elevation of AFP at diagnosis in patients with pre-existing chronic liver condition suggests the utility of surveillance based on serum AFP measurement alone. This is further supported by a multi-center survey in Japan based on 548 children with chronic HBV infection, where 15 (2.7%) patients were eventually diagnosed with HCC during childhood or young adulthood with regular AFP measurement (24). Devising such surveillance scheme requires screening for vaccine failure in children born to mothers who are HBV carriers. This may address the pitfall of HBV vaccination, especially for males who appear to be especially vulnerable to development of HCC during childhood based on our experience and previous epidemiological studies (11). A guideline incorporating schedule for universal HBV vaccination, post-vaccination serologic testing to identify vaccination failure as well as a surveillance scheme for children with chronic HBV infection will be the keys in reducing the risk and improving the outcome of pediatric HCC in HBV endemic regions.

There are several limitations to our study. First, due to the rarity of pediatric HCC and the retrospective nature of our study, our cohort size was small and the treatment approach was heterogeneous, precluding further subgroup or multivariable analyses. Nonetheless, the inclusion of all patients from two developed HBV endemic regions over two decades allowed us to verify previously identified prognostic factors and explore the impact of chronic HBV infection in the real world setting where patients are managed with curative intent. Second, imaging and tumor material from earlier patients were not available for review thus limiting our capability to perform full retrospective staging or central pathological review. Lastly, virological analyses were restricted to serum HBV surface antigen and antibody. Since HBe seroconversion has been associated with the onset of HCC, the inclusion of HBe antigen, anti-HBe antibody, and HBV DNA levels in future studies will allow better correlation between the timing for tumor development and host immune response (24).

In conclusion, pediatric HCC is associated with an aggressive clinical course and complete resection offers the best chance of cure. HBV-associated pediatric HCC carries a worse prognosis but may be mitigated by implementation of surveillance strategies.

## Data Availability Statement

The original contributions presented in the study are included in the article/**Supplementary Material**, further inquiries can be directed to the corresponding author.

## Ethics Statement

The studies involving human participants were reviewed and approved by The University of Hong Kong/Hong Kong West Cluster, Hong Kong, KK Women’s and Children’s Hospital, Singapore. Written informed consent from the participants’ legal guardian/next of kin was not required to participate in this study in accordance with the national legislation and the institutional requirements.

## Author Contributions

All authors were involved in the data curation and writing—reviewing and editing. ALi, SYS, ALo, and AChi were involved in the conceptualization, methodology, and writing of the original draft. ALi, HP was in charge of the data analysis, methodology, and visualization. SYS, ALo, and AChi supervised the study. All authors contributed to the article and approved the submitted version.

## Funding

The study was supported by the VIVA Foundation for Children with Cancer (ALo, SYS).

## Conflict of Interest

The authors declare that the research was conducted in the absence of any commercial or financial relationships that could be construed as a potential conflict of interest.

## Supplementary Material

Supplementary Figure 1 Relationship between patient’s HBV serology, maternal HBV serology and HBV vaccination. HBsAg, hepatitis B surface antigen; mat, maternal; NA, not available, pt, paternal.

Supplementary Table 1 Demographics, clinical characteristics, treatment and outcome of the study cohort.

Supplementary Table 2 Demographics, biochemical, radiographical characteristics according to study region.
