# Supplementary figures and images for: Hepatitis B Virus Seropositivity Is a Poor Prognostic Factor of Pediatric Hepatocellular Carcinoma: a Population-Based Study in Hong Kong and Singapore
Source: Front Oncol. 2020 Nov 20;10:570479. doi: 10.3389/fonc.2020.570479 (PMC7716753; doi:10.3389/fonc.2020.570479)

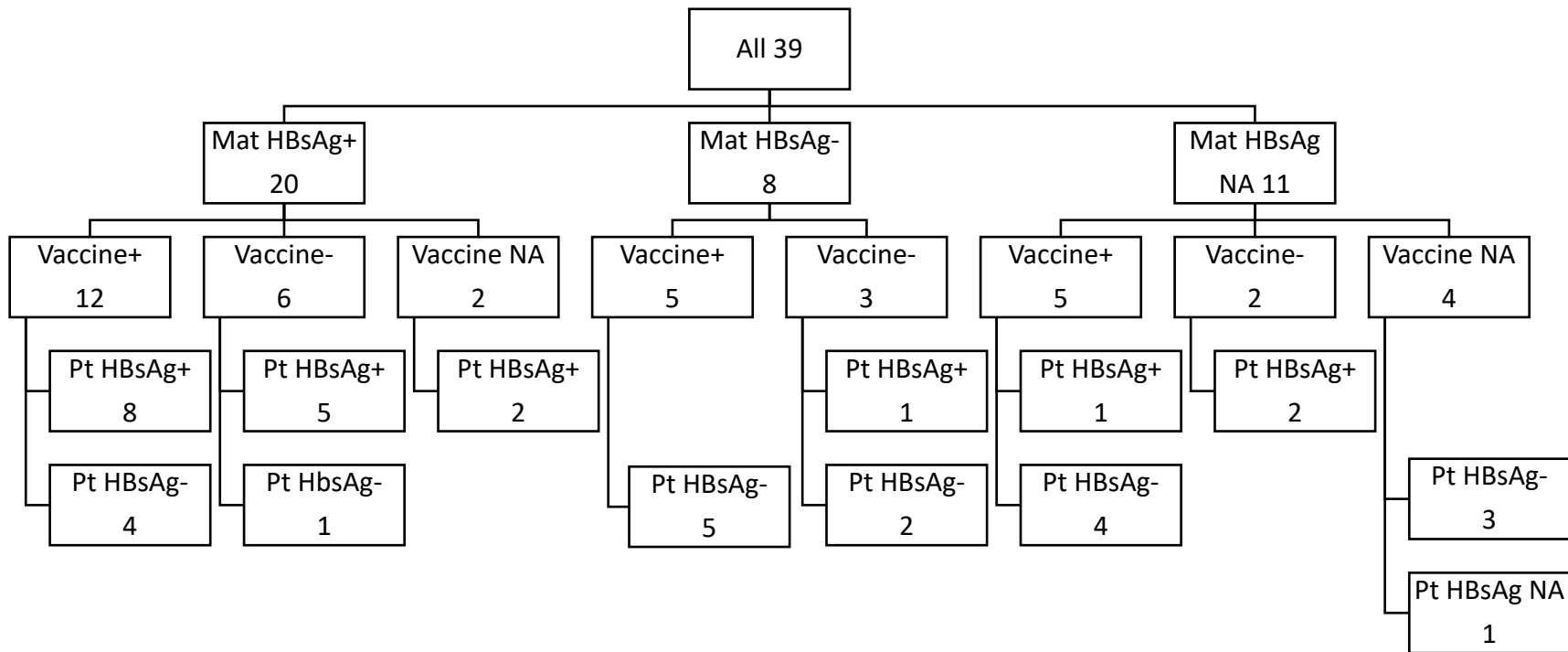

Supplement: Supplementary Figure 1 — Relationship between patient’s HBV serology, maternal HBV serology and HBV vaccination. HBsAg, hepatitis B surface antigen; mat, maternal; NA, not available, pt, paternal. [file Image_1.pdf]
