# Supplementary material for: Hepatitis B Virus Seropositivity Is a Poor Prognostic Factor of Pediatric Hepatocellular Carcinoma: a Population-Based Study in Hong Kong and Singapore
Source: Front Oncol. 2020 Nov 20;10:570479. doi: 10.3389/fonc.2020.570479 (PMC7716753; doi:10.3389/fonc.2020.570479)
Supplement: Supplementary Table 2 — Demographics, biochemical, radiographical characteristics according to study region. [file Table_2.docx]

|  | Entire cohort | | Hong Kong | | Singapore | | P value |
| --- | --- | --- | --- | --- | --- | --- | --- |
|  | **N** | **%** | **N** | **%** | **N** | **%** |  |
| Total | 39 | 100 | 28 | 100 | 11 | 100 |  |
|  |  |  |  |  |  |  |  |
| Sex |  |  |  |  |  |  | 1 |
| Male | 30 | 77 | 21 | 75 | 9 | 82 |  |
| Female | 9 | 23 | 7 | 25 | 2 | 18 |  |
|  |  |  |  |  |  |  |  |
| Age of diagnosis (years) (mean±SD) | 10.2  ±4.1 | - | 10.7  ±4.0 | - | 8.9  ±4.0 | - | 0.208 |
|  |  |  |  |  |  |  |  |
| Patient HBsAg |  |  |  |  |  |  | 0.713 |
| Positive | 19 | 49 | 15 | 54 | 4 | 36 |  |
| Negative | 19 | 49 | 13 | 46 | 6 | 55 |  |
| NA | 1 | 3 | 0 | 0 | 1 | 9 |  |
|  |  |  |  |  |  |  |  |
| Period of diagnosis |  |  |  |  |  |  | 1 |
| 1993-2001 | 19 | 49 | 14 | 50 | 5 | 46 |  |
| 2002-2017 | 20 | 51 | 14 | 50 | 6 | 55 |  |
|  |  |  |  |  |  |  |  |
| ALP (IU/L)  (mean±SD) | 267  ±195 | - | 280  ±210 | - | 212  ±102 | - | 0.264 |
| ALT (IU/L)  (mean±SD) | 287  ±1151 |  | 91  ±86 |  | 947  ±2401 |  | 0.347 |
| AFP (ng/ml)  (mean±SD) | 500598  ±1350405 | - | 652868  ±1551061 | - | 74244  ±74283 | - | 0.059 |
|  |  |  |  |  |  |  |  |
| Lobe involvement |  |  |  |  |  |  | 1 |
| Left | 6 | 15 | 5 | 18 | 1 | 9 |  |
| Right | 11 | 28 | 8 | 29 | 3 | 27 |  |
| Bilateral | 19 | 49 | 14 | 50 | 5 | 45 |  |
| NA | 3 | 8 | 1 | 4 | 2 | 18 |  |
|  |  |  |  |  |  |  |  |
| Portal vein involvement |  |  |  |  |  |  | 1 |
| Yes | 7 | 18 | 5 | 18 | 2 | 18 |  |
| No | 27 | 69 | 19 | 68 | 8 | 73 |  |
| NA | 5 | 13 | 4 | 14 | 1 | 9 |  |
|  |  |  |  |  |  |  |  |
| Distant metastasis |  |  |  |  |  |  | 0.725 |
| Yes | 17 | 44 | 13 | 46 | 4 | 36 |  |
| No | 20 | 51 | 14 | 50 | 6 | 55 |  |
| NA | 2 | 5 | 1 | 4 | 1 | 9 |  |
|  |  |  |  |  |  |  |  |
| Regional lymph node |  |  |  |  |  |  | 0.557 |
| Involved | 4 | 10 | 4 | 14 | 0 | 0 |  |
| Not involved | 33 | 85 | 23 | 82 | 10 | 91 |  |
| NA | 2 | 5 | 1 | 4 | 1 | 9 |  |
|  |  |  |  |  |  |  |  |
| Definitive resection |  |  |  |  |  |  | 0.458 |
| Yes | 16 | 41 | 11 | 39 | 5 | 45 |  |
| No | 21 | 54 | 17 | 61 | 4 | 36 |  |
| NA | 2 | 5 | 0 | 0 | 2 | 18 |  |

*AFP, alpha fetoprotein; ALP, alkaline phosphatase; HBsAg, hepatitis B surface antigen; NA, not available; SD, standard deviation*

**Supplementary Table S2. Demographics, biochemical, radiographical characteristics according to study region.**
